# Blood Cancer Network Ireland (BCNI) and National Cancer Registry Ireland (NCRI) collaboration: challenges and utility of an Enhanced Blood Cancer Outcomes Registry (EBCOR) pilot

**DOI:** 10.1007/s11845-024-03756-9

**Published:** 2024-07-20

**Authors:** Seán R. Millar, Mohamed Bakri Mohamed, Vitaliy Mykytiv, Rose McMorrow, Conan Donnelly, Eamonn O’Leary, Nina Orfali, Philip Murphy, Paul V. Browne, John Quinn, Peter O’Gorman, Mary F. Ryan, Ruth Clifford, Ezzat El Hassadi, Derville O’Shea, Oonagh Gilligan, Janusz Krawczyk, Michael E. O’Dwyer, Eva Szegezdi, Mary R. Cahill

**Affiliations:** 1 School of Public Health, University College Cork, Cork, Ireland; 2 Haematology Department, Cork University Hospital, Cork, Ireland; 3 National Cancer Registry Ireland (NCRI), Cork, Ireland; 4 International Niemann-Pick Disease Registry, Newcastle, UK; 5 Haematology Department, St James’s Hospital, Dublin, Ireland; 6 Haematology Department, Beaumont Hospital, Dublin, Ireland; 7 Haematology Department, The Mater Misericordiae University Hospital, Dublin, Ireland; 8 Haematology Department, University Hospital Limerick, Limerick, Ireland; 9 Haematology Department, University Hospital Waterford, Waterford, Ireland; 10 Haematology Department, University Hospital Galway, Galway, Ireland; 11 University of Galway, Galway, Ireland

**Keywords:** Acute myeloid leukaemia, Blood cancers, Cancer registry, Charlson comorbidity index, Prediction, Survival

## Abstract

**Background:**

The Blood Cancer Network Ireland and National Cancer Registry Ireland worked to create an Enhanced Blood Cancer Outcomes Registry (EBCOR). Enhanced data in acute myeloid leukaemia (AML) included an extensive data dictionary, bespoke software and longitudinal follow-up.

**Aims:**

To demonstrate the utility of the database, we applied the data to examine a clinically relevant question: Charlson comorbidity index (CCI) usefulness in predicting AML patients’ survival.

**Methods:**

A software designer and consultant haematologists in Cork University Hospital worked together to standardise a data dictionary, train registrars and populate a database. One hundred and forty-one AML patients underwent enhanced data registration. Comorbidities identified by chart review were used to examine the capability of the CCI and age at diagnosis to predict mortality using Kaplan–Meier curves, Cox regression and receiver operating characteristic curves.

**Results:**

In regression analysis, a dose–response relationship was observed; patients in the highest CCI tertile displayed a greater risk (HR = 4.90; 95% CI 2.79–8.63) of mortality compared to subjects in tertile 2 (HR = 2.74; 95% CI 1.64–4.57) and tertile 1 (reference). This relationship was attenuated in an analysis which adjusted for age at diagnosis. The area under the curve (AUC) for the CCI was 0.76 (95% CI 0.68–0.84) while the AUC for age at diagnosis was 0.84 (95% CI 0.78–0.90).

**Conclusions:**

Results suggest that the CCI provides no additional prognostic information beyond that obtained from age alone at AML diagnosis and that an EBCOR can provide a rich database for cancer outcomes research, including predictive models and resource allocation.

**Supplementary Information:**

The online version contains supplementary material available at 10.1007/s11845-024-03756-9.

## Introduction

**Fig. 1:**
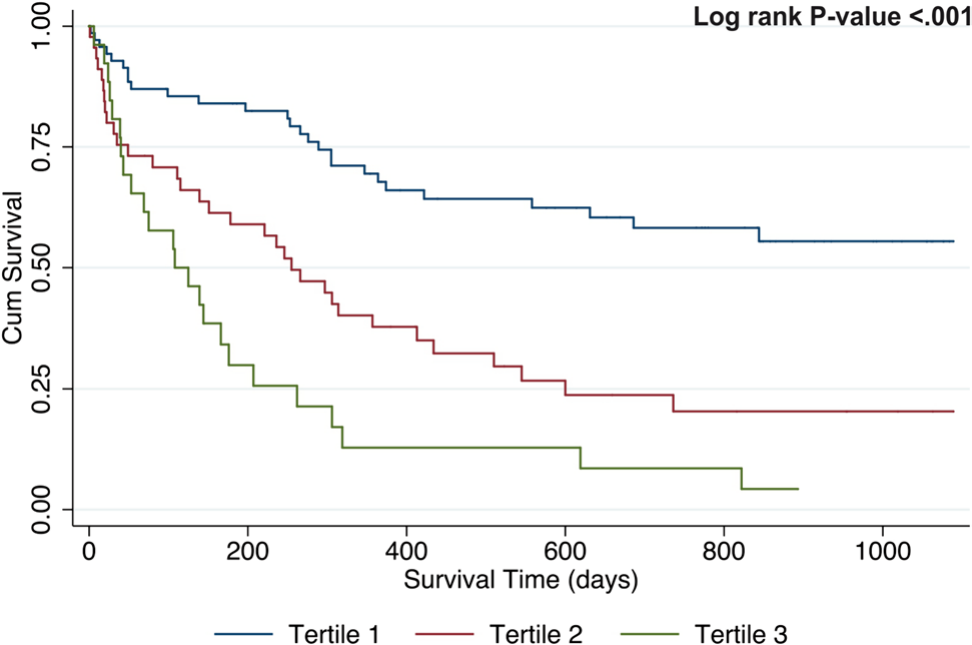
Acute myeloid leukaemia survival in respect to Charlson comorbidity index tertiles. The figure shows Kaplan–Meier curves for CCI tertiles to predict survival among AML patients. The median survival time for patients in the lowest tertile of the CCI was 498.5 days, compared to 246.0 and 116.5 days for subjects in tertiles 2 and 3, respectively

**Fig. 2:**
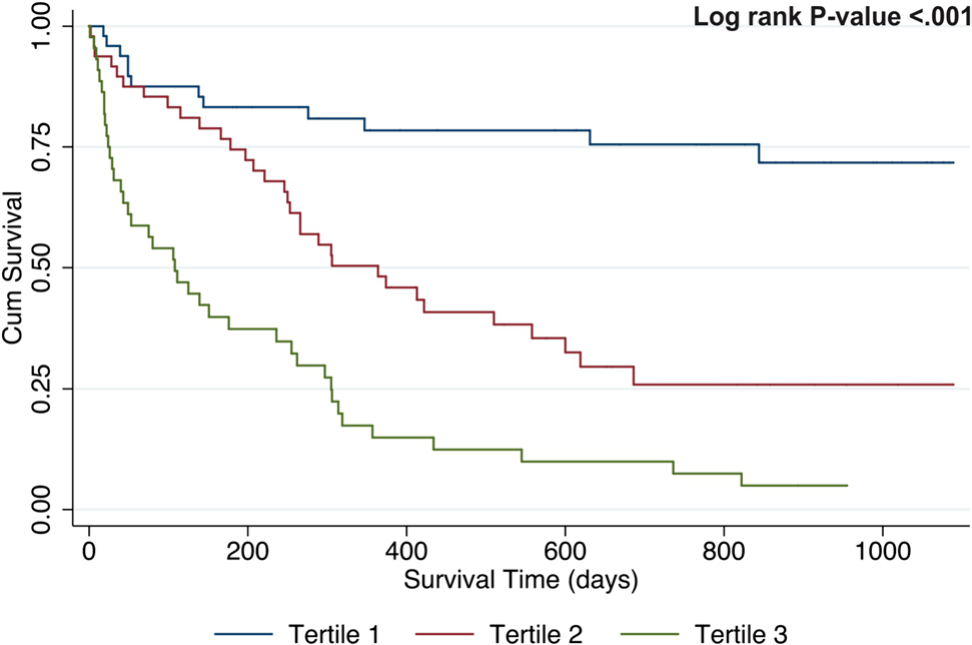
Acute Myeloid Leukaemia survival in respect to age at diagnosis tertiles. The figure shows Kaplan–Meier curves for age at diagnosis tertiles to predict survival among AML patients. The median survival time for patients in the lowest age tertile was 765.0 days, compared to 305.5 and 107.0 days for subjects in tertiles 2 and 3, respectively

Cancer registration is time-consuming, even in countries with well organised electronic records. In Ireland, data are published with lag times of around 24 months [1]. Currently, the National Cancer Registry Ireland (NCRI) collect basic registration data on complex cancers such as acute myeloid leukaemia (AML) and report a crude AML incidence in Ireland of 3.0 per 100,000 population for 2017–2019, which is broadly in line with European figures [1, 2]. Blood cancers require complex diagnostic and classificatory tests, intensive therapy (associated with significant bed usage for treatment and management of adverse effects) and prolonged transfusion and antibiotic support for associated bone marrow failure [3]. Consequently, AML is a costly malignancy to treat. However, the diagnostic and treatment costs associated with AML have not been evaluated in Ireland. Studies in the UK found that costs varied according to treatment intensity, amounting to £112,545 for patients who receive intensive chemotherapy followed by allogenic stem cell transplant compared to £3708 for those receiving best supportive care [4]. For the NCRI to be in a position to evaluate AML outcomes and costs, detailed data on diagnostics and therapy would need to be collected [5–8].

The Blood Cancer Network Ireland (BCNI) secured funding from the Science Foundation Ireland (SFI) and the Irish Cancer Society (ICS) for research in partnership with the NCRI to explore the setting up of enhanced data registration—an Enhanced Blood Cancer Outcomes Registry (EBCOR)—of blood cancers in Ireland [9]. To date, there is little published on population-based EBCOR by cancer registries. Publications are, in the main, on clinical registries as opposed to population-based registries. One example is the National Gynae-Oncology Registry (NGOR) in Monash, Australia, where the published protocol paper details some of the difficulties with ensuring coverage of all incident cases and with data collection [10]. In Ireland, the NCRI operates on a statutory basis and consent is not required. Monash registries operate on an ‘opt out’ consent model and not by statute [11–13].

In our pilot, collaboration between haematologists, the BCNI and NCRI was designed to maximise the chances of a successful project, shorten registration time for blood cancers and enhance the quality and amount of data collected. The challenges of this project were considerable and included extensive training of data registrars in order to provide knowledge on rapid developments in diagnostics and changes in classification of blood cancers, expansion of useful diagnostic datasets during the study period and fragmentation of patients’ clinical case notes (both electronic and paper based). The potential benefits of enhanced data in AML include having an organised extensive database, the ability to contribute to international datasets, greater capacity to identify clinical trial–eligible patients, the ability to ask and answer prognostic-relevant and economic questions and the addition of longitudinal follow-up data within the NCRI.

The prognosis of AML depends on patient and disease characteristics; patient factors include age, general condition and social factors. Disease factors are well documented and include AML subtype, blast count, genetics, cytogenetics and full blood counts [5–8]. In addition, older adult patients with AML often have a substantial comorbidity burden. Consequently, comorbidity scores may be used in clinical practice to help quantify multiple comorbidities. The Charlson comorbidity index (CCI), calculated based on 19 different medical conditions, weighs the comorbidities to measure a patient’s additional burden of disease, over and above the issues of AML [14]. Although there are publications suggesting that the CCI may be useful for assessing survival in AML patients [15–17], and that its use could help determine which older patients would benefit from more intensive treatment, the CCI is cumbersome to calculate and is not in routine use in Ireland for AML prognostic assessment.

The Irish EBCOR pilot aimed to show that enhanced blood cancer registration, rolled out nationally, could provide data on which to improve the selection of patients who might benefit from intensive treatment. This could help reduce the side effect burden which falls disproportionately on older adults, reduce the financial costs of treating AML and assist in national healthcare planning. Therefore, drawing on data from 141 AML patients who underwent enhanced data registration, we applied the CCI to the AML-enhanced dataset to examine a simple, but clinically relevant, question: Is the CCI useful for predicting survival in AML patients?

## Materials and methods

### Study design and population

Research funded data registrars and a software designer worked with consultant haematologists in University College Cork’s (UCC) Cork University Hospital (CUH) to plan and implement a data dictionary of enhanced data in AML, relevant not just to diagnosis, but to treatment and prognosis as well. Data registrars in the NCRI are drawn from diverse backgrounds, including nursing, and undergo training in identification and classification of incident cancer cases. Solid tumours are identified on biopsy by histopathologists and coded using Systematised Nomenclature of Medicine (SNOMED) codes. SNOMED is a standardised, international, multilingual core set of clinical healthcare terminology, suitable for use in electronic records. Data registrars are also trained in using International Classification of Diseases (ICD) codes. However, blood cancers, which comprise around 10% of cancers, are not always identified on a tissue biopsy, and sometimes a bone marrow liquid aspirate, flow cytometry or pathognomonic genetic test is used to make the diagnosis. This presents a more diverse diagnostic landscape for data registrars to navigate, and more extensive training was required.

Consultants and laboratory staff in CUH identified new AML patients in real time, notified NCRI staff and assisted with ensuring that all diagnostic material was captured in a bespoke database, designed for the study by the NCRI software designer. The EBCOR study registrars underwent extensive training in the nature of AML, diagnostic and classificatory procedures and received on-site assistance from the consultant haematologists. Registrar training included World Health Organization classifications of AML, use of the laboratory electronic data system —APEX, assessment of flow cytometry, karyotypic and genetic reports and further training on the appraisal of patients’ paper record charts. Additional instruction on the identification and recording of complications and comorbidity was provided with attention to relevant validated scales, such as the CCI and Eastern Cooperative Oncology Group (ECOG)/Karnofsky scores [18]. The NCRI research manager and analytic staff had extensive input to this process.

The design and pilot of the project took place over 24 months, from January 1st 2017 to December 31st 2019. An external haematology consultant-led audit was carried out on sample registrations to validate the completeness and quality of the AML data. Further changes were implemented and then the enhanced collection in real time was rolled out nationally to four major leukaemia treating centres. The research funding was obtained from the SFI and the ICS as part of a linked project to connect early phase clinical trials in blood cancers with biobanking and data collection within the BCNI network. In total, 141 AML patients underwent enhanced data registration and were followed until death, emigration or the 31st of December 2019. Data were obtained under the NCRI statute which does not require patient consent; all collected data were fully anonymised and we had no access to information that could identify individual participants during or after data collection. Ethics committee approval to conduct this study was obtained from the Clinical Research Ethics Committee of UCC.

### Data collection

The electronic AML-specific data collection instrument and data dictionary used for the EBCOR contains 164 items on the AML patients, including data on AML subtype, genetics and cytogenetics, blood counts and transfusion as well as information on sex, age, marital status, medical insurance, treatment details, comorbidities and survival. The system used was designed and built for the project and consisted of drop-down menus with tick box acceptance for the main diagnostic criteria. Free text was avoided. For rare occurrences, there was an ‘Other’ option; for example, under ‘Karyotype’, the menu contained all of the major described abnormalities as a defined selectable option. However, the ‘Genetic abnormalities’ menu required frequent revision and free text was considered to allow for rapidly developing advances in diagnosis and classification.

The selection of variables for the data dictionary was dictated by current best practice and international standardisation [6]. We liaised with colleagues in Monash University in Australia who were also working on an EBCOR at that time. Data registrars, based between the NCRI and the hospitals involved, underwent training for the task which was delivered by consultant haematologists. Data collection involved examination of paper hospital notes and relevant electronic databases such as APEX, Lantis, Aria, NCIS, PIMS and IMPAX in use for treatment scheduling and administration. Laboratory data were extracted from hospital laboratory information systems and the NCRI was given remote access to CUH’s laboratory information system to expedite and facilitate the set-up of the project. Quality control and data audits were carried out by consultants on pilot data and retraining and refinement of data collection was applied as needed. The electronic data collection sheet was modified following pilot use. Data registrars had real time access to consultant advice on coding and data interpretation. Following initial stages in CUH, the pilot was rolled out to a further three hospitals treating AML in Galway and Dublin, with overall coverage estimated at 33% of total national incidence of AML in Ireland for the years 2017–2019.

### Charlson comorbidity index (CCI) calculation

Comorbidities were assessed by chart review using ICD-9 codes. Identified comorbidities were given a weighted score; a score of 1 for myocardial infarction, congestive heart failure, peripheral vascular disease, cerebrovascular disease, dementia, chronic pulmonary disease, connective tissue disease, ulcer disease, mild liver disease and diabetes. A score of 2 was assigned to each of the following comorbidities: hemiplegia, moderate or severe renal failure, diabetes with end organ damage, any tumour, leukaemia and lymphoma; a score of 3 was assigned to moderate or severe liver disease while a score of 6 was assigned to metastatic solid tumours and AIDS. A combined age–comorbidity score was then calculated [14].

### Treatment

For the purpose of this study, we categorised patients, depending on the treatment they received, into three mutually exclusive groups: intensive treatment, non-intensive treatment/non-curative treatment and supportive treatment only. Intensive treatment includes all patients who underwent myeloablative inpatient cytotoxic chemotherapy regimens that are classically prescribed for fitter patients with curative intention such as high-dose cytarabine, anthracycline or both, in different combinations, sometimes with the addition of other drugs [8]. These regimens exclusively necessitate hospitalisation for administration and this group includes all patients who received at least one cycle of intensive treatment. The group treated with non-curative intent comprised of patients who received inpatient or outpatient low-intensity regimens (hydroxycarbamide, azacitidine, decitabine, venetoclax and low-dose cytarabine). The supportive treatment only group encompassed patients who received symptomatic supportive treatment and blood products, with no systemic anticancer therapy.

### Statistical analysis

Complete data for the CCI calculation and age at diagnosis were available for all subjects (*n* = 141). Descriptive characteristics were examined for the full sample and according to CCI and age at diagnosis tertiles. Categorical features are presented as percentages. The CCI and age are shown as a median and interquartile range. Differences across CCI and age tertiles were analysed using chi-square tests. Kaplan–Meier curves, Cox regression and time-dependent receiver operating characteristic (ROC) curves were used to determine the ability of the CCI and age at diagnosis to predict mortality among AML patients. Data analysis was conducted using Stata SE Version 13 (Stata Corporation, College Station, TX, USA) for Windows. For all analyses, a *P*-value (two-tailed) of less than 0.05 was considered to indicate statistical significance.

## Results

### Descriptive characteristics

Table 1 shows characteristics of patients at diagnosis with AML included in this study. There were 141 patients diagnosed with AML, aged 17–94 years, from the Irish EBCOR between 2017 and 2019. The median age of patients was 68 (interquartile range: 54–77), with 44.7% of patients being 70 years of age or older; a majority (55.5%) were married and over one-third (37.3%) had private medical insurance. Almost half (47.5%) and over one-quarter (28.4%) of patients had undergone intensive treatment or non-intensive/non-curative treatment, respectively; for 17.7% of patients, treatment status was unknown.

**Table 1:** Characteristics of patients at diagnosis with acute myeloid leukaemia (*n* = 141)

Characteristic	Level	Value
Sex	Male vs. female	85 (60.3)
Age at diagnosis	Median	68 (54–77)
	< 50	26 (18.4)
	50–69	52 (36.9)
	> 70	63 (44.7)
Charlson comorbidity index	Median	3.0 (1.0–4.0)
Marital status	Married	76 (55.5)
	Single	22 (16.1)
	Divorced/separated/widowed	19 (13.9)
	Unknown	20 (14.6)
Private medical insurance	Yes vs. no	47 (37.3)
Treatment	Intensive treatment	67 (47.5)
	Non-intensive/non-curative treatment	40 (28.4)
	Supportive treatment only	9 (6.4)
	Treatment unknown	25 (17.7)

Numbers and percentages (in parentheses) are shown. Age at diagnosis (continuous) and Charlson comorbidity index are shown as a median and (interquartile range)

When examining characteristics across CCI and age tertiles (Supplementary Table 1), it was observed that patients in the lowest CCI tertile, and younger patients, were more likely to be married and less likely to be divorced/separated or widowed. Differences in treatment level were also noted, with patients in the lower CCI and age tertiles being more likely to have undergone intensive treatment compared to patients in the highest tertile categories, who were more likely to have received non-intensive/non-curative treatment only. A scatterplot of the CCI and age at diagnosis is shown in Supplementary Fig. 1. The *r* value was 0.612, indicating that the CCI and age at diagnosis were moderately correlated.

### Survival

Figure 1 and Fig. 2 present Kaplan–Meier survival curves for the CCI and age at diagnosis tertiles. Of the 141 AML patients, 84 had died by 31/12/2019 (median survival time = 289.0 days). For both the CCI and age, there were statistically significant differences when comparing the survival curves (log rank *P*-value < 0.001, for both). The median survival time for patients in the lowest tertile of the CCI was 498.5 days, compared to 246.0 and 116.5 days for subjects in tertiles 2 and 3, respectively. The median survival time for patients in the lowest age tertile was 765.0 days, compared to 305.5 and 107.0 days for subjects in tertiles 2 and 3, respectively.

### Cox regression

Hazard ratios (HR) for the CCI and age at diagnosis to predict AML mortality, as both linear (continuous) and categorical variables, are displayed in Table 2. In univariate analyses, a dose–response relationship was observed, with patients in the highest CCI tertile (HR = 4.90; 95% CI 2.79–8.63) and highest age tertile (HR = 8.36; 95% CI 4.33–16.15) displaying a greater risk of mortality compared to subjects in tertile 2 (HR = 2.74; 95% CI 1.64–4.57 and HR = 3.62; 95% CI 1.85–7.06, for the CCI and age, respectively) and tertile 1 (reference). However, in an analysis which adjusted for age at diagnosis, the relationship between the CCI and mortality was attenuated and non-significant (model 4). In contrast, age at diagnosis remained significantly associated with mortality in adjusted models. In sensitivity analyses (not shown), which excluded patients whose treatment status was unknown, the same relationships were observed.

**Table 2:** Cox regression models for the Charlson comorbidity index and age at diagnosis to predict acute myeloid leukaemia mortality

	Model 1		Model 2		Model 3		Model 4		Model 5	
	HR (95% CI)	*P*-value	HR (95% CI)	*P*-value	HR (95% CI)	*P*-value	HR (95% CI)	*P*-value	HR (95% CI)	*P*-value
Charlson comorbidity index										
Linear	1.15 (1.09–1.22)	< 0.001	1.15 (1.08–1.22)	< 0.001	1.07 (1.00–1.15)	0.07	1.01 (0.93–1.10)	0.753	0.98 (0.88–1.09	0.716
Tertile 1	1.00 (Ref.)	< 0.001	1.00 (Ref.)	< 0.001	1.00 (Ref.)	0.008	1.00 (Ref.)	0.293	1.00 (Ref.)	0.532
Tertile 2	2.74 (1.64–4.57)		2.72 (1.63–4.53)		1.91 (1.06–3.44)		0.86 (0.45–1.64)		0.80 (0.36–1.79)	
Tertile 3	4.90 (2.79–8.63)		4.71 (2.63–8.45)		2.92 (1.49–5.72)		1.31 (0.64–2.72)		1.12 (0.44–2.81)	
Age at diagnosis										
Linear	1.07 (1.05–1.09)	< 0.001	1.07 (1.05–1.09)	< 0.001	1.06 (1.03–1.08)	< 0.001	1.07 (1.04–1.09)	< 0.001	1.06 (1.03–1.09)	< 0.001
Tertile 1	1.00 (Ref.)	< 0.001	1.00 (Ref.)	< 0.001	1.00 (Ref.)	< 0.001	1.00 (Ref.)	< 0.001	1.00 (Ref.)	0.002
Tertile 2	3.62 (1.85–7.06)		3.39 (1.72–6.66)		3.14 (1.57–6.28)		3.56 (1.79–7.11)		2.78 (1.27–6.06)	
Tertile 3	8.36 (4.33–16.15)		8.26 (4.27–15.96)		5.47 (2.50–12.00)		8.08 (3.73–17.50)		6.01 (2.19–16.46)	

Hazard ratios (HR) and 95% confidence intervals (CI) are shown

Model 1: univariate

Model 2: adjusted for sex

Model 3: adjusted for treatment

Model 4: adjusted for each other

Model 5: adjusted for sex, treatment, each other, marital status and private medical insurance

No significant interaction between the CCI and age was noted when both were examined as continuous variables (*P*-value = 0.379). We additionally tested an interaction between the CCI as a continuous variable and age as a categorical (below median age/above median age) variable in a fully adjusted model. The *P*-value for the test of interaction was significant at 0.001, suggesting effect modification, with a plot of the interaction effect showing the CCI to have poor predictive ability among AML patients who were older (Supplementary Fig. 2).

### ROC analysis

**Fig. 3:**
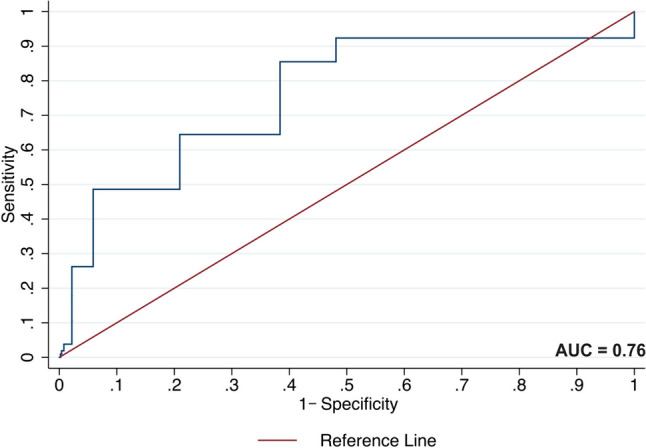
Time-dependent ROC curve for the Charlson comorbidity index to predict acute myeloid leukaemia mortality. The figure shows an ROC curve for the CCI to predict mortality among AML patients. The AUC for the CCI was 0.76 (95% CI 0.68–0.84)

**Fig. 4:**
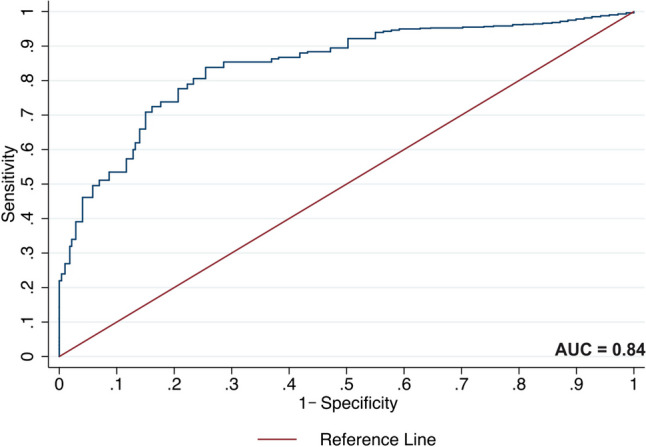
Time-dependent ROC curve for age at diagnosis to predict acute myeloid leukaemia mortality. The figure shows an ROC curve for age at diagnosis to predict mortality among AML patients. The AUC for age at diagnosis was 0.84 (95% CI 0.78–0.90)

Time-dependent ROC curves for the CCI and age at diagnosis to predict mortality are shown in Fig. 3 and Fig. 4. The area under the curve (AUC) provides a scale from 0.5 to 1 (with 0.5 representing random chance and 1 indicating perfect discrimination) by which to appraise the capacity of the CCI and age to predict mortality. The AUC for the CCI was 0.76 (95% CI 0.68–0.84), suggesting only moderate discriminatory ability. The AUC for age at diagnosis was 0.84 (95% CI 0.78–0.90), indicating a good ability to predict mortality among AML patients.

As the interaction effect between the CCI as a continuous variable and age at diagnosis as a categorical variable suggested effect modification, we conducted additional sensitivity analyses stratifying our sample using a median age cut-off (data not shown). Among AML patients less than 68 years of age, the AUC for age at diagnosis to predict mortality was found to be 0.67 (95% CI 0.53–0.81) while the AUC for the CCI was 0.63 (95% CI 0.49–0.77). Among patients aged 68 years or older, the AUC for age at diagnosis was also 0.67 (95% CI 0.51–0.83) while the AUC for the CCI was 0.51 (95% CI 0.32–0.70), indicating little to no ability to predict mortality among older AML patients.

## Discussion

In this study, we describe the process of an EBCOR in AML and examine the usefulness of the CCI to predict AML survival, drawing on data from 141 AML patients who underwent extended data registration as part of the EBCOR pilot in Ireland. Although univariate analysis suggested a dose–response relationship between the CCI and AML survival, this association was attenuated in an analysis which adjusted for age at diagnosis. In addition, when compared to age at AML diagnosis, the CCI demonstrated only moderate discriminatory ability to predict AML mortality. Consequently, these results suggest that the CCI provides no additional prognostic information beyond that which is obtained from age alone at AML diagnosis.

Ultimately, the aim of this study was to pilot a system that could be developed and rolled out nationally in a sustainable manner. While this aim was not achieved, the pilot has provided a rich database for researching AML treatment and outcomes in an Irish setting. The process of enhanced data registration as applied to a complex cancer, such as AML, proved difficult, resource-intensive and requiring of upskilled data registrars. Within CUH, the NCRI data registrars were embedded as part of the haematology team. However, the application outside the pilot centre in CUH, despite the central training of the registrars, was less successful than anticipated. The success of a future EBCOR in Ireland will be heavily dependent on it being embedded into core work of the cancer registry, as well as close cooperation with clinical cancer teams on the ground. In addition, the sustainability of an EBCOR depends on trained staff to capture complex data. Thus, staffing and associated costs of the registry would need to increase to allow for this, as once grant funding ceased, the data registrar posts for trained EBCOR data collectors also ceased.

We also learned that harmonisation of data recording systems in the national cancer centres in Ireland is essential for a future EBCOR, as each hospital has different electronic systems, laboratory procedures and processes for data handling. Barriers to enhanced data collection included, *inter alia*, the disparate notes that comprise cancer patients’ records. These varied between the hospitals involved (a source of additional difficulty in standardisation) and included paper and electronic records on a variety of different systems. This problem prevents Irish cancer researchers from being maximally involved in international data consortia [19]. The National Cancer Control Programme (NCCP) are currently addressing the issue of data harmonisation by rolling out a unified electronic system to cancers centres in Ireland. Our research suggests that this should be beneficial in future recording of blood cancers.

There is some literature published discussing the difficulty of implementing an EBCOR in blood cancers. The successful Australian registries on bone marrow failure syndromes, plasma cell disorders, lymphoma, haemoglobinopathies and blood transfusion, publish regular reports [20] and acknowledge dependence on clinician commitment and participation. A recent Australian study noted difficulties in ensuring full data capture and collection [10]. In Ireland, haematologists recognise the lag in NCRI reporting and also the need to improve capture of blood cancers, which are sometimes diagnosed using different methods, and which may not always receive a SNOMED code, as solid tumours do. This makes case ascertainment more difficult for the NCRI data registration staff. In this EBCOR pilot, we succeeded in reducing the registration time to under a month, potentially allowing for up-to-date analysis in a field where therapy is rapidly changing, and more older patients are undergoing treatment.

Importantly, our findings confirm that enhanced data registration can be used to test and answer useful questions which may include resource allocation and best targeting of care to patients. We examined the utility of the complex CCI which, as expected, is predictive of mortality in our dataset. Univariate analysis showed the CCI to be strongly associated with AML survival, but this relationship was attenuated in a model which adjusted for age at diagnosis. This attenuation was also found by Bouligny et al. in their cohort of patients treated with hypomethylating agents and venetoclax [17]. In addition, we found that when compared to age at AML diagnosis, the CCI demonstrated only moderate discriminatory ability to predict AML mortality, with sensitivity analyses showing the CCI to be poor at predicting mortality among those who were older, findings which have been previously observed [21]. Consequently, despite some studies indicating that the comorbidity burden measured by the CCI independently predicts early mortality and outcome survival in patients with AML [15, 16], our data suggest that the CCI provides no additional prognostic information beyond that which is obtained from age alone at AML diagnosis. This finding is important (calculating the CCI is labour-intensive and time-consuming due to diverse patient records) and relevant (as the upper age for treatment continues to rise).

In an era where sophisticated risk stratification, based on karyotype and genetics, is usual practice, the striking utility of age alone is a reminder of the covert biological variables that are inevitably present with age. Nonetheless, age alone should never be the primary deciding factor in AML treatment decisions. Age at AML diagnosis in combination with cytogenetic/molecular data, in addition to comprehensive geriatric assessments which incorporate functional, cognitive and socioeconomic factors, is more optimal in prognostication/treatment selection [22]. Further advances in the electronic records for patients with cancers in Ireland will allow automatic calculation of algorithms for other prognostic indices [22, 23], and their comparison, once fields are populated.

Our research provides the first EBCOR database in Ireland to allow studies such as appraisal of the CCI and age at diagnosis as predictors of AML survival. The availability of effective, less-intensive day ward-based treatments, such as azacitidine combined with venetoclax, and target oral therapies such as midostaurin [24–27], make an EBCOR and the assessment of AML outcomes more relevant than ever. However, this study has several limitations. These include the small number of patients available for analysis, which did not include all newly diagnosed AML patients in Ireland during the study period. In addition, the BCNI/NCRI enhanced registration dataset has missing variables for some patients. For future patients, data collection will improve. As previously discussed, a national database is currently being introduced and piloted in Ireland by the NCCP. The data fields in our database will help provide a consistent, reproducible and auditable data resource for future monitoring of blood cancer outcomes and treatment effects in Ireland.

In conclusion, we show that although implementing an EBCOR is difficult and resource demanding, it can provide useful data for answering questions on which future resource allocation (of both time and treatment resources) can be rationally decided. The exigencies of an EBCOR for a complex blood cancer, such as AML, should not be underestimated and such a national database would facilitate participation with international initiatives such as the European Union Harmony Alliance [28], with whom the BCNI already has links. However, the requirements to initiate, maintain and analyse such data include dedicated and trained staff, harmonisation of data collection and ongoing quality control. This study provides a template for the training needs of staff undertaking data entry, management and quality control in an EBCOR. The work of the NCCP and All-Island eHealth-Hub for cancer [19] will further advance matters regarding data harmonisation and data quality for cancer research in Ireland.

## Supplementary Information

Below is the link to the electronic supplementary material.

Supplementary file1 (DOCX 35 KB)

## Data Availability

For information on how to access the Irish EBCOR data, please contact Professor Mary Cahill, Email: mary.cahill@ucc.ie**/**MaryR.Cahill@hse.ie.
