# Supplementary material for: Blood Cancer Network Ireland (BCNI) and National Cancer Registry Ireland (NCRI) collaboration: challenges and utility of an Enhanced Blood Cancer Outcomes Registry (EBCOR) pilot
Source: Ir J Med Sci. 2024 Jul 20;193(6):2615–23. doi: 10.1007/s11845-024-03756-9 (PMC11666745; doi:10.1007/s11845-024-03756-9)
Supplement: Supplementary file 1 — Supplementary file1 (DOCX 35 KB) [file 11845_2024_3756_MOESM1_ESM.docx]

**Supplementary Table 1.** Characteristics of patients at diagnosis with Acute Myeloid Leukaemia according to Charlson Comorbidity Index and age at diagnosis tertiles

| **Characteristic** | **Level** | **Charlson Comorbidity Index tertiles** | | | |
| --- | --- | --- | --- | --- | --- |
|  |  | **Tertile 1** | **Tertile 2** | **Tertile 3** | **P-value** |
| **Sex** | Male vs. female | 39 (55.7) | 27 (60.0) | 19 (73.1) | .303 |
| **Marital status** | Married | 41 (60.3) | 23 (52.3) | 12 (48.0) | .016* |
|  | Single | 13 (19.1) | 3 (6.8) | 6 (24.0) |  |
|  | Divorced/separated/widowed | 3 (4.4) | 11 (25.0) | 5 (20.0) |  |
|  | Unknown | 11 (16.2) | 7 (15.9) | 2 (8.0) |  |
| **Private medical insurance** | Yes vs. no | 20 (34.5) | 17 (38.6) | 10 (41.7) | .808 |
| **Treatment** | Intensive treatment | 49 (70.0) | 14 (31.1) | 4 (15.4) | <.001* |
|  | Non-intensive/non-curative treatment | 7 (10.0) | 17 (37.8) | 16 (61.5) |  |
|  | Supportive treatment only | 3 (4.3) | 4 (8.9) | 2 (7.7) |  |
|  | Treatment unknown | 11 (15.7) | 10 (22.2) | 4 (15.4) |  |
| **Characteristic** | **Level** | **Age at diagnosis tertiles** | | | |
|  |  | **Tertile 1** | **Tertile 2** | **Tertile 3** | **P-value** |
| **Sex** | Male vs. female | 25 (51.0) | 36 (75.0) | 24 (54.5) | .035 |
| **Marital status** | Married | 29 (61.7) | 30 (65.2) | 17 (38.6) | .001 |
|  | Single | 10 (21.3) | 8 (17.4) | 4 (9.1) |  |
|  | Divorced/separated/widowed | 3 (6.4) | 2 (4.3) | 14 (31.8) |  |
|  | Unknown | 5 (10.6) | 6 (13.0) | 9 (20.5) |  |
| **Private medical insurance** | Yes vs. no | 15 (36.6) | 18 (40.9) | 14 (34.1) | .807 |
| **Treatment** | Intensive treatment | 38 (77.6) | 26 (54.2) | 3 (6.8) | <.001* |
|  | Non-intensive/non-curative treatment | 4 (8.2) | 14 (29.2) | 22 (50.0) |  |
|  | Supportive treatment only | 1 (2.0) | 2 (4.2) | 6 (13.6) |  |
|  | Treatment unknown | 6 (12.2) | 6 (12.5) | 13 (29.5) |  |

Numbers and percentages (in parentheses) are shown.

*P-value computed using Fisher’s Exact Test as cells have an expected count less than 5.


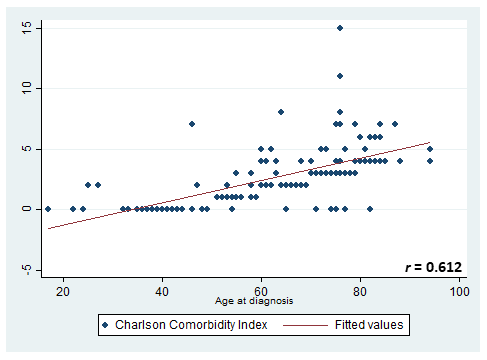


**Supplementary Fig. 1** Scatterplot of the Charlson Comorbidity Index and age at diagnosis

The figure shows a scatterplot of the CCI and age at diagnosis. The *r* value was 0.612, indicating that the CCI and age at diagnosis were moderately correlated.


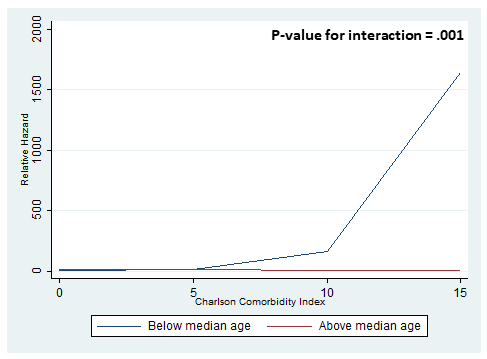


**Supplementary Fig. 2** Interaction plot of predicted Acute Myeloid Leukaemia mortality by the Charlson Comorbidity Index and age at diagnosis

The figure shows a plot of the interaction effect between the CCI as a continuous variable and age at diagnosis as a categorical variable (below median age/above median age). The P-value for the test of interaction was significant at .001, suggesting effect modification.
